# Pan-immune inflammation value and neutrophil-to-albumin ratio predict hemorrhagic transformation after intravenous thrombolysis in acute ischemic stroke: a dual-center cohort study

**DOI:** 10.3389/fnut.2026.1724969

**Published:** 2026-05-18

**Authors:** Zhuang Zhu, Yao Geng, Hualin Wang, Bo Du, Min Xu, Kefan Yi, Jiaxin Shi, Xufeng Wang, Yongsheng Yuan, Deqin Geng, Kezhong Zhang

**Affiliations:** 1 Department of Neurology, The First Affiliated Hospital with Nanjing Medical University, Nanjing, Jiangsu, China; 2 The First School of Clinical Medicine, Nanjing Medical University, Nanjing, Jiangsu, China; 3 Department of Neurology, The Affiliated Hospital of Xuzhou Medical University, Xuzhou, Jiangsu, China; 4 Clinical Nutrition Department, Shanghai Deji Hospital, Qingdao University, Shanghai, China

**Keywords:** acute ischemic stroke, hemorrhagic transformation, intravenous thrombolysis, neutrophil-to-albumin ratio, pan-immune inflammation value

## Abstract

**Background and purpose:**

Hemorrhagic transformation (HT) remains a critical complication of intravenous thrombolysis (IVT) in acute ischemic stroke (AIS), yet reliable pre-thrombolysis predictors are limited. We investigated whether pan-immune inflammation value (PIV) and neutrophil-to-albumin ratio (NAR), individually and in combination, are associated with HT and its subtypes.

**Methods:**

In this dual-center retrospective cohort study, 4,313 AIS patients treated with IVT were included. PIV and NAR were calculated from routine baseline blood tests. The primary outcome was any HT, with secondary outcomes including ECASS-classified subtypes: hemorrhagic infarction (HI1, HI2) and parenchymal hematoma (PH1, PH2). Associations were evaluated using multivariable logistic regression, restricted cubic spline (RCS) modeling, subgroup analyses, and receiver operating characteristic (ROC) curves.

**Results:**

Hemorrhagic transformation patients exhibited significantly higher PIV and NAR than non-HT patients, with the highest levels in PH1 and PH2. Multivariable analyses identified both PIV and NAR as independent predictors of overall HT, PH1, and PH2. Joint high PIV and NAR conferred a markedly increased risk (OR = 6.63, 95% CI: 4.78–9.17). Subgroup analyses confirmed the consistency of associations across age, sex, baseline NIHSS, and comorbidities. RCS modeling demonstrated a logarithmic non-linear relationship between these inflammatory markers and HT risk (non-linearity *p* < 0.05). ROC analysis showed that combining PIV and NAR substantially improved predictive accuracy (AUC = 0.783, 95% CI: 0.761–0.804, *p* < 0.001) compared to individual markers.

**Conclusion:**

Elevated PIV and NAR are independently associated with increased risk of HT, particularly PH1 and PH2, after IVT in AIS patients. Integrating these biomarkers enhances predictive performance, supporting their potential utility in multidimensional risk stratification before thrombolysis.

## 1 Introduction

Acute ischemic stroke (AIS) is the leading cause of disability and the second most common cause of death worldwide, accounting for approximately 80–85% of all strokes and nearly 5.5 million deaths annually (1). Despite advances in reperfusion therapy, including endovascular thrombectomy, intravenous recombinant tissue plasminogen activator (rt-PA) thrombolysis remains the cornerstone of acute treatment. Large randomized trials have shown that rt-PA significantly increases the likelihood of functional independence, with an absolute benefit of 12–16% when administered within 4.5 h (2). Nevertheless, the therapeutic benefits of rt-PA are offset by the risk of hemorrhagic transformation (HT), which is observed radiologically in up to 40% of cases and occurs symptomatically in 6–8% (3). Severe HT, especially parenchymal hematoma, is associated with high mortality and worsened functional prognosis (4), making accurate risk prediction an urgent unmet need.

Traditionally, research on HT pathophysiology has focused on vascular factors, such as reperfusion injury and blood–brain barrier (BBB) breakdown. More recently, attention has shifted to the interplay between systemic inflammation, immune dysregulation, and nutritional status. Stroke induces a rapid systemic inflammatory response characterized by neutrophil infiltration, platelet activation, and monocyte-driven immune cascades, all of which amplify BBB disruption and secondary injury (5, 6). Elevated inflammatory markers, such as the neutrophil-to-lymphocyte ratio (NLR), have been independently linked with HT and unfavorable outcomes (7). In parallel, malnutrition—affecting up to 40% of stroke patients—has been identified as a modifiable determinant of poor recovery, increasing the risk of infection, functional decline, and long-term mortality (8, 9). Importantly, immune-inflammatory activation and nutritional depletion are not isolated events; rather, they are intricately linked through a vicious biological cycle (10). On one hand, severe systemic inflammation triggers a hypercatabolic state, increasing metabolic demand and accelerating protein degradation, thereby precipitating or worsening malnutrition (11). On the other hand, nutritional depletion—particularly hypoalbuminemia—deprives the neurovascular unit of crucial antioxidant, anti-inflammatory, and endothelial-stabilizing defenses. Consequently, an unchecked neutrophil-driven inflammatory response, coupled with diminished nutritional resilience, creates a synergistic “double hit” on the BBB (12). This interplay ultimately exacerbates BBB disruption, profoundly increasing the patient’s vulnerability to HT after thrombolysis.

Several clinical scores, including Hemorrhage After Thrombolysis (HAT) score (13), Symptomatic Intracranial Hemorrhage after Stroke Thrombolysis (SEDAN) score (14), and Stroke Prognostication using Age and NIHSS (SPAN-100) score (15), have been proposed for predicting HT. However, these models are limited by their reliance on neuroimaging or complex calculations, restricting their bedside applicability. In this context, simple and integrated biomarkers reflecting both systemic inflammation and nutritional status may provide greater clinical utility.

The pan-immune-inflammation value (PIV) has recently gained attention as a multidimensional marker derived from neutrophil, platelet, monocyte, and lymphocyte counts (16). Unlike single-ratio indices, PIV incorporates multiple immune cell subsets, thereby capturing both pro-inflammatory and regulatory immune components (17). Previous studies have validated PIV as a prognostic marker in oncology and cardiovascular disease (18–20), but its relevance to AIS, particularly to post-thrombolysis HT, remains unexplored.

Similarly, the neutrophil-to-albumin ratio (NAR) directly captures this complex interplay between systemic immune activation and nutritional status. Elevated neutrophils represent acute inflammatory activation, while reduced albumin reflects impaired vascular integrity and diminished antioxidant capacity (21, 22). NAR has been associated with outcomes in traumatic brain injury, subarachnoid hemorrhage, and malignancies (23, 24), but its predictive role in AIS and HT has not yet been investigated.

Therefore, this dual-center cohort study aimed to examine the independent and synergistic associations of PIV and NAR with HT and its ECASS-defined subtypes in AIS patients receiving intravenous rt-PA. By focusing on immune-inflammatory and nutritional markers, we sought to provide novel insights into the immunological mechanisms underlying HT and propose clinically accessible biomarkers for early risk stratification in thrombolyzed patients.

## 2 Objects and methods

### 2.1 Study design and population

This multicenter retrospective observational study enrolled AIS patients who received intravenous rt-PA thrombolysis at the First Affiliated Hospital of Nanjing Medical University and the Affiliated Hospital of Xuzhou Medical University from February 2018 to August 2025.

Inclusion criteria required meeting all of the following:

1. Age ≥18 years;
2. AIS confirmed by CT/MRI imaging;
3. Onset-to-treatment time window ≤4.5 h;
4. Standard rt-PA intravenous thrombolysis protocol (total dose 0.9 mg/kg, maximum ≤90 mg: initial 10% bolus over 1 min, remaining 90% infused over 60 min);
5. Complete clinical data and informed consent from patients or legal representatives.

Inclusion criteria required meeting all of the following:

1. Exclusion criteria included:

1. Patients receiving bridging therapy (intravenous thrombolysis combined with endovascular intervention or intra-arterial thrombolysis) (*n* = 52);
2. Severe cardiorenal dysfunction, coagulation disorders, history of substance abuse, or hemodynamic instability (*n* = 26);
3. Chronic liver/kidney failure or chronic heart failure (*n* = 14);
4. Systemic inflammatory/infectious diseases, hematological disorders, or immunosuppressive therapy within 2 weeks pre-admission (*n* = 76);
5. Comorbidities potentially affecting inflammatory biomarkers (malignancy, acute myocardial infarction, major trauma, recent surgery, or allergic diseases) (*n* = 44);
6. Incomplete hospitalization records or missing data impacting follow-up assessments (*n* = 127).

**Figure 1:**
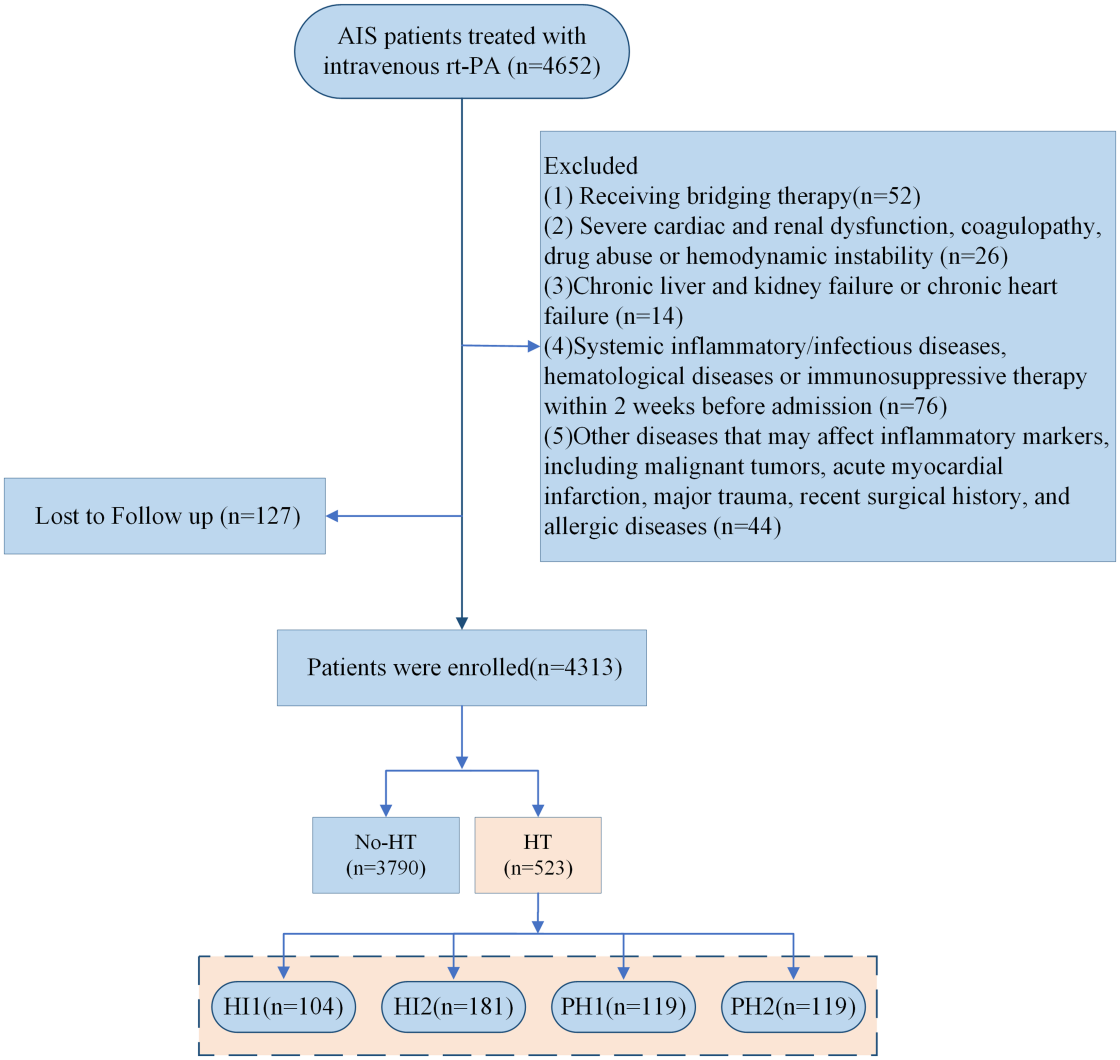
Flowchart of patient selection. AIS, acute ischemic stroke; rt-PA, recombinant tissue plasminogen activator; HT, hemorrhagic transformation; HI1, hemorrhagic infarction type 1; HI2, hemorrhagic infarction type 2; PH1, parenchymal hematoma type 1; PH2, parenchymal hematoma type 2.

The study strictly adhered to the ethical principles of the Declaration of Helsinki and was approved by the Ethics Committees of the First Affiliated Hospital with Nanjing Medical University (Approval No.: 2025-SR-414) and the Affiliated Hospital of Xuzhou Medical University (Approval No.: XYFY2018-KL038-01). Informed consent was obtained from all participants or their legal representatives. The patient screening process is illustrated in Figure 1.

### 2.2 Methods

#### 2.2.1 Data collection and definition

Two trained neurologists collected the data. All data were collected prior to IV-tPA administration. We collected demographic characteristics, vascular risk factors, medical history, infarction distribution (anterior circulation, posterior circulation, or both), laboratory data, imaging information, and treatment details (including interventions before and after AIS onset). BMI was calculated as weight (kg) divided by height squared (m^2^) (25). Hypertension, diabetes, atrial fibrillation, and coronary heart disease were defined according to World Health Organization (WHO) criteria. Stroke subtypes were classified using the Trial of Org 10,172 in Acute Stroke Treatment (TOAST) classification (26). Alberta Stroke Program Early CT Score (ASPECTS) was evaluated from admission brain non-contrast-enhanced CT images.

All venous blood samples, including complete blood count (CBC), biochemical tests (including albumin and admission blood glucose), and coagulation function, were collected upon admission and strictly prior to IV-tPA administration, according to standard institutional guidelines (27, 28). Additionally, inflammatory parameters derived from CBC were calculated. NLR = neutrophil count/lymphocyte count, PIV = (neutrophil count × platelet count × monocyte count)/lymphocyte count, NAR = neutrophil count/albumin level.

#### 2.2.2 Outcome assessment

Primary outcome was hemorrhagic transformation (HT), defined as a new hemorrhage confirmed by imaging (CT/MRI) within 24 h after intravenous thrombolysis, with no evidence of hemorrhage on pre-thrombolysis imaging (29, 30).

**Figure 2:**
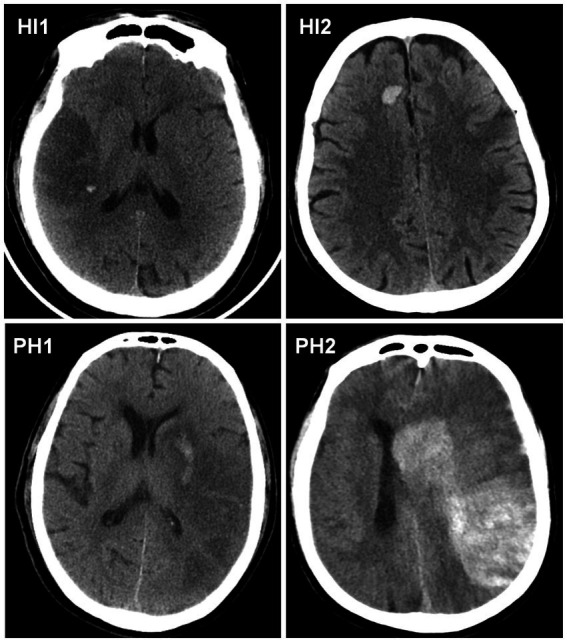
CT scan examples of hemorrhagic infarction type 1 (HI1), HI2, parenchymal hematoma type 1 (PH1) and PH2, according to the European Cooperative Acute Stroke Study criteria. HI1, hemorrhagic infarction type 1; HI2, hemorrhagic infarction type 2; PH1, parenchymal hematoma type 1; PH2, parenchymal hematoma type 2.

Secondary outcome was the classification of HT according to the ECASS criteria (30, 31). Two blinded neuroradiologists categorized HT into four subtypes: hemorrhagic infarction type 1 (HI1, petechial hemorrhage at infarct margins), HI2 (confluent petechiae within the infarct without mass effect), parenchymal hematoma type 1 (PH1, hematoma volume <30% of infarct area with mild mass effect), and PH2 (hematoma volume >30% of infarct area or significant mass effect). Representative CT images of the HT subtypes are presented in Figure 2.

#### 2.2.3 Statistical analysis

The normality of continuous variables was assessed using the Shapiro–Wilk test. Data with a normal distribution were presented as mean ± standard deviation (*x̄* ± *s*) and compared using independent-sample *t*-tests (binary outcomes) or one-way ANOVA (multiclass outcomes). Non-normally distributed data were presented as median [interquartile range, M(Q25, Q75)] and analyzed with the Mann–Whitney *U* test (binary outcomes) or Kruskal–Wallis *H* test (multiclass outcomes). Categorical variables were expressed as frequency (%) and compared using Pearson’s *χ*^2^ test or Fisher’s exact test. For multiple comparisons, Bonferroni correction was applied. To approximate a normal distribution, PIV and NAR were log2-transformed prior to analysis. Both continuous and categorical forms of PIV and NAR were evaluated; when categorized, they were divided into quartiles (Q1–Q4) according to their distribution in the study population.

Potential confounders associated with stroke prognosis were first screened by univariate analysis. Subsequently, multivariable logistic regression models were constructed to assess the independent and joint associations of PIV and NAR with HT and HT subtypes in AIS patients, considering both prior literature and clinical relevance (32–34). Three models were specified: Model 1, unadjusted; Model 2, adjusted for age and sex; and Model 3, further adjusted for smoking, hypertension, atrial fibrillation, prior stroke or TIA, infarct distribution, baseline NIHSS score, ASPECTS, blood glucose (BG), onset-to-needle time (ONT), antiplatelet therapy, anticoagulant therapy, TOAST classification, and LDL.

Additive and multiplicative interactions between PIV and NAR on HT were evaluated. Additive interactions were quantified by calculating relative excess risk due to interaction (RERI), attributable proportion (AP), and synergy index (SI) with 95% CIs, using variance estimators from previous studies (35). Multiplicative interactions were tested by including PIV, NAR, and their product term in the regression model. Restricted cubic spline (RCS) regression with four knots was applied to explore potential nonlinear associations between PIV, NAR, and HT, with threshold effect analyses used to identify inflection points.

To further investigate effect modification, subgroup analyses were conducted. Based on theoretical considerations and prior studies, prespecified stratification variables included sex, age, smoking, drinking, hypertension, atrial fibrillation, diabetes, coronary heart disease, previous stroke or TIA, NIHSS, and TOAST classification. Stratified regression analyses were performed to examine the consistency of associations between PIV, NAR, HT, and HT subtypes across subgroups.

The predictive performance of PIV, NAR, and their combination for HT was assessed by calculating the area under the receiver operating characteristic (ROC) curve (AUC). Additionally, two other metrics, the net reclassification index (NRI) and integrated discrimination improvement (IDI), were calculated to assess the incremental predictive value of PIV, NAR, and their combination. All analyses were performed using IBM SPSS Statistics version 26.0, R version 4.3.1, and MedCalc version 20.1, with two-tailed *p* < 0.05 considered statistically significant.

## 3 Results

Of the 4,313 included patients, 64.8% were male (*n* = 2,794), with a median age of 68 years (IQR 59–76). The HT rate was 12.1% (523/4313). HT subtypes were distributed as follows: HI1 2.4% (104/4313), HI2 4.2% (181/4313), PH1 2.8% (119/4313), and PH2 2.8% (119/4313).

### 3.1 Baseline characteristics of primary outcomes

**Figure 3:**
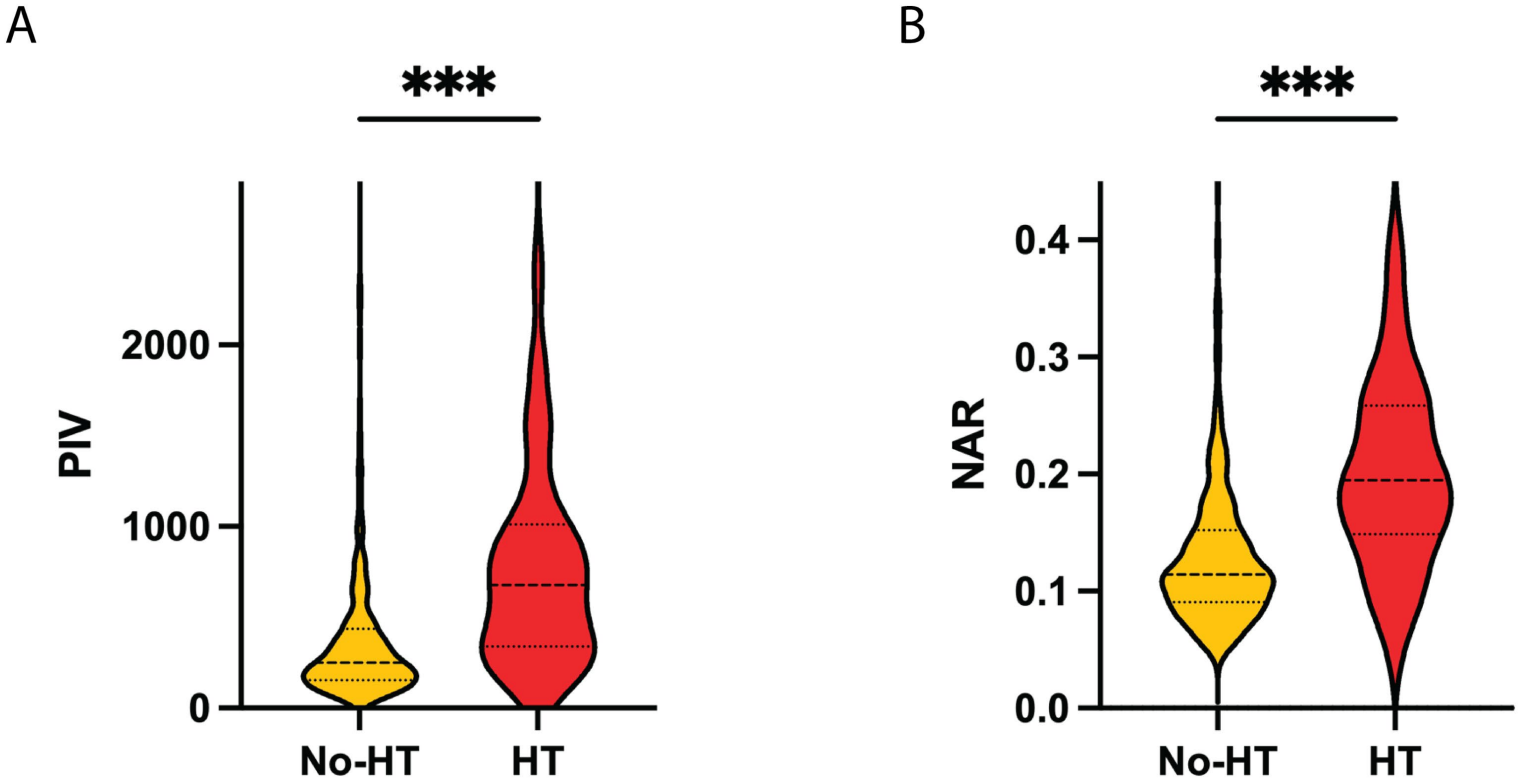
Distribution of PIV and NAR levels between HT and non-HT groups. **(A)** Violin plot displaying the distribution of PIV values, showing significantly higher levels in the HT group compared to the No-HT group. **(B)** Violin plot displaying the distribution of NAR values, also showing a significant increase in the HT group. ***P < 0.001. HT, hemorrhagic transformation; PIV, pan-immune inflammation value; NAR, neutrophil-to-albumin ratio.

Compared with the non-HT group, patients with HT had significantly higher baseline NIHSS scores, longer onset-to-needle time, higher blood pressure, and elevated levels of BG, homocysteine, hs-CRP, NLR, PIV, and NAR. They were also more likely to have atrial fibrillation, smoking, drinking, and prior anticoagulant use, with significant differences in TOAST classification and infarct distribution. In contrast, patients in the HT group had a lower prevalence of hypertension and prior use of antiplatelet, statin, or antihypertensive therapy, and lower ASPECTS (all *p* < 0.05) (Figure 3 and Table 1).

**Table 1:** Comparison of baseline data between the HT and non-HT groups.

Variable	Overall (*n* = 4,313)	HT (*n* = 523)	Non-HT (*n* = 3,790)	*P*
Demographic characteristics				
Male, *n* (%)	2,794 (64.78)	340 (65.01)	2,454 (64.75)	0.907
Age, years	68 (59, 76)	71 (62, 79)	68 (59, 76)	<0.001
BMI, Kg/m^2^	24.80 (22.80, 27.20)	25.10 (22.50, 27.20)	24.80 (22.86, 27.20)	0.991
Risk factors, *n* (%)				
Smoking history	1,050 (24.35)	148 (28.30)	902 (23.80)	0.025
Drinking history	1,124 (26.06)	163 (31.17)	961 (25.36)	0.005
Hypertension	2,889 (66.98)	294 (56.21)	2,595 (68.47)	<0.001
Diabetes	1,156 (26.80)	145 (27.72)	1,011 (26.68)	0.612
Atrial fibrillation	789 (18.29)	218 (41.68)	571 (15.07)	<0.001
Previous stroke or TIA	1,194 (27.68)	124 (23.71)	1,070 (28.23)	0.030
CHD	712 (16.51)	96 (18.36)	616 (16.25)	0.225
Medical history, *n* (%)				
Prior antiplatelet	496 (11.50)	40 (7.65)	456 (12.03)	0.003
Prior anticoagulant	86 (1.99)	25 (4.78)	61 (1.61)	<0.001
Prior statin	393 (9.11)	28 (5.35)	365 (9.63)	0.001
Antihypertensive	1,514 (35.10)	156 (29.83)	1,358 (35.83)	0.007
Prior hypoglycemic	599 (13.89)	72 (13.77)	527 (13.91)	0.932
Clinical variables				
NIHSS, score	7 (4, 12)	15 (9, 21)	6.00 (4, 11)	<0.001
ASPECTS, score	9 (8, 10)	7 (6, 8)	9 (8, 10)	<0.001
ONT, minute	165 (111, 219)	185 (133, 240)	164 (111, 215)	<0.001
SBP, mmHg	152 (139, 168)	156 (143, 168)	152 (139, 168)	0.002
DBP, mmHg	86 (78, 96)	87 (79, 99)	86 (78, 95)	0.001
Infarct distribution, *n* (%)				<0.001
Anterior circulation	2,203 (51.08)	231 (44.17)	1,972 (52.03)	
Posterior circulation	1,134 (26.29)	59 (11.28)	1,075 (28.36)	
Anterior and posterior circulation	976 (22.63)	233 (44.55)	743 (19.60)	
TOAST, *n* (%)				<0.001
LAA	1,774 (41.13)	279 (53.35)	1,495 (39.45)	
CE	546 (12.66)	154 (29.45)	392 (10.34)	
SAO	1,629 (37.77)	49 (9.37)	1,580 (41.69)	
Others	364 (8.44)	41 (7.84)	323 (8.52)	
Laboratory data				
BG, mmol/L	6.11 (5.04, 6.89)	6.59 (5.89, 9.27)	6.01 (5.01, 6.67)	<0.001
Homocysteine, μmol/L	16.28 (12.40, 17.50)	17.04 (13.50, 17.04)	15.99 (12.32, 17.69)	<0.001
LDL, mmol/L	2.66 (2.16, 3.12)	2.66 (1.98, 2.99)	2.66 (2.18, 3.17)	0.002
HDL, mmol/L	1.09 (0.91, 1.20)	1.10 (0.96, 1.19)	1.08 (0.91, 1.20)	0.250
Hs-CRP, mg/L	2.20 (0.60, 7.10)	4.40 (0.80, 13.00)	2.00 (0.60, 6.00)	<0.001
NLR	3.22 (2.12, 5.38)	6.86 (4.17, 11.32)	3.02 (2.04, 4.54)	<0.001
PIV	277.20 (165.29, 508.46)	676.67 (340.46, 1,010.43)	249.67 (154.01, 436.17)	<0.001
NAR	0.12 (0.09, 0.17)	0.19 (0.15, 0.26)	0.11 (0.09, 0.15)	<0.001

HT, hemorrhagic transformation; BMI, Body Mass Index; TIA, transient ischemic attack; CHD, Coronary heart disease; NIHSS, National Institutes of Health Stroke Scale; ONT, Onset-to-Needle Time; SBP, systolic blood pressure; DBP, diastolic blood pressure; TOAST, Trial of Org 10,172 in Acute Stroke Treatment; LAA, large-artery atherosclerosis; CE, cardioembolism; SAO, small-artery occlusion; BG, blood glucose; LDL, low-density lipoprotein; HDL, high-density lipoprotein; Hs-CRP, hyper-sensitive C-reactive protein; NLR, neutrophil-to-lymphocyte ratio; PIV, pan-immune inflammation value; NAR, neutrophil-to-albumin ratio.

### 3.2 Baseline characteristics of secondary outcomes

**Figure 4:**
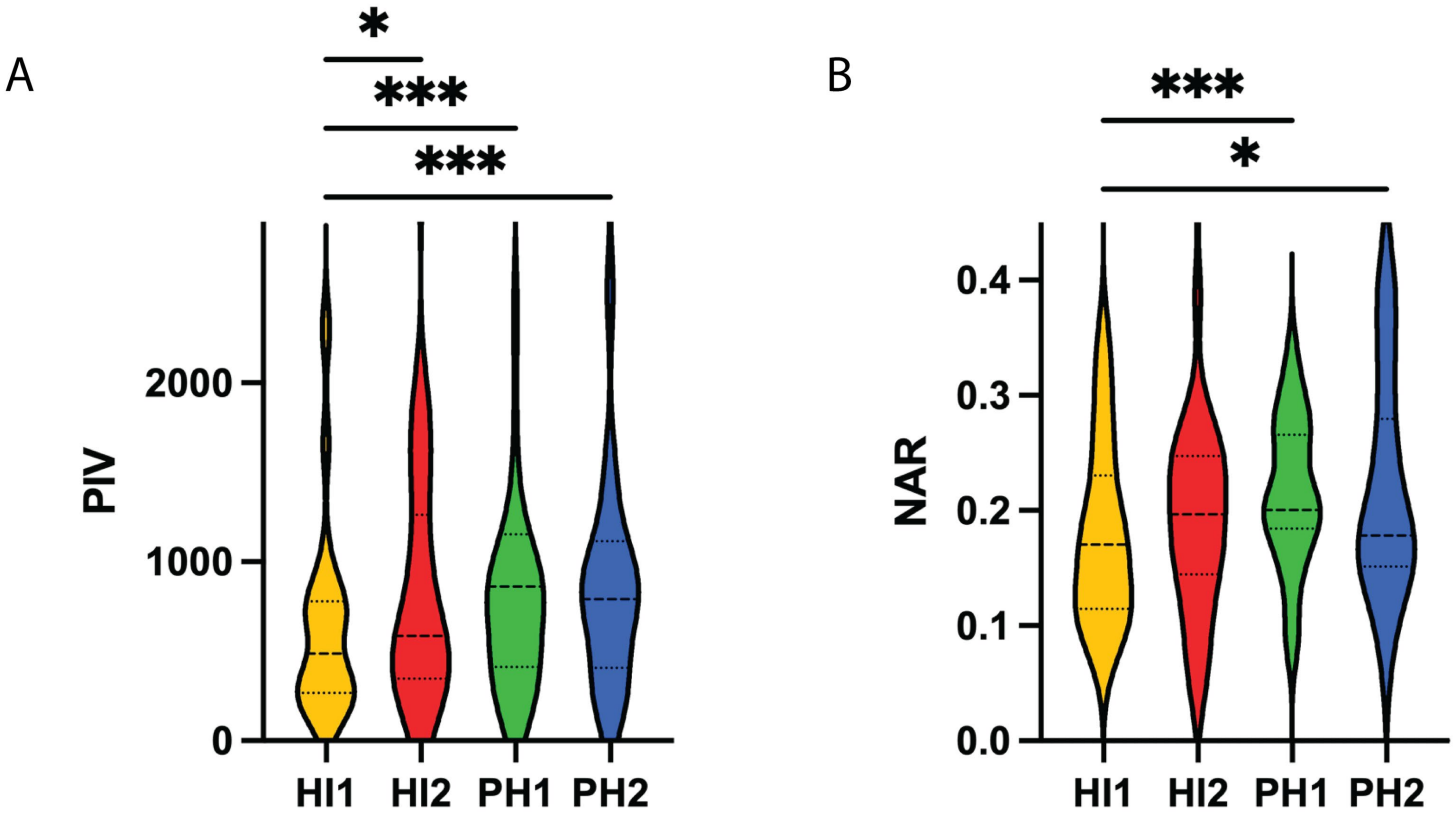
Distribution of PIV and NAR levels across different HT subtypes. **(A)** Violin plot comparing PIV values among HI1, HI2, PH1, and PH2 groups. **(B)** Violin plot comparing NAR values among the four HT subtypes. Significant differences between groups are indicated by asterisks. *P < 0.05, ***P < 0.001. HI1, hemorrhagic infarction type 1; HI2, hemorrhagic infarction type 2; PH1, parenchymal hematoma type 1; PH2, parenchymal hematoma type 2; PIV, pan-immune inflammation value; NAR, neutrophil-to-albumin ratio.

Among HT patients, baseline characteristics varied significantly across subtypes. PH1 and PH2 patients had higher NIHSS scores, NLR, and PIV levels, and lower ASPECTS, suggesting more severe neurological deficits and systemic inflammation, while HI1 patients had the highest prevalence of hypertension, diabetes, atrial fibrillation, and prior antihypertensive or hypoglycemic therapy. Infarct distribution and TOAST classification also differed among subtypes, with LAA most common in HI1 and CE in PH1 and PH2, reflecting distinct clinical and laboratory profiles across HT subtypes (Figure 4 and Table 2).

**Table 2:** Comparison of baseline characteristics among HI-1, HI-2, PH-1, and PH-2 groups.

Variable	Overall (*n* = 523)	HI1 (*n* = 104)	HI2 (*n* = 181)	PH1 (*n* = 119)	PH2 (*n* = 119)	*P*
Demographic characteristics						
Male, *n* (%)	340 (65.01)	70 (67.31)	114 (62.98)	94 (78.99)	62 (52.10)	<0.001
Age, years	71 (62, 79)	69 (58, 74)	74 (68, 79)	72 (64, 80)	64 (56, 78)	0.001
BMI, Kg/m^2^	25.10 (22.50, 27.20)	26.55 (23.90, 28.76)	25.18 (22.50, 26.00)	23.70 (20.70, 25.80)	25.18 (23.60, 28.90)	<0.001
Risk factors, *n* (%)						
Smoking history	148 (28.30)	13 (12.50)	39 (21.55)	53 (44.54)	43 (36.13)	<0.001
Drinking history	163 (31.17)	28 (26.92)	40 (22.10)	53 (44.54)	42 (35.29)	<0.001
Hypertension	294 (56.21)	86 (82.69)	96 (53.04)	50 (42.02)	62 (52.10)	<0.001
Diabetes	145 (27.72)	47 (45.19)	40 (22.10)	38 (31.93)	20 (16.81)	<0.001
Atrial fibrillation	218 (41.68)	29 (27.88)	96 (53.04)	57 (47.90)	36 (30.25)	<0.001
Previous stroke or TIA	124 (23.71)	36 (34.62)	38 (20.99)	27 (22.69)	23 (19.33)	0.031
CHD	96 (18.36)	19 (18.27)	39 (21.55)	19 (15.97)	19 (15.97)	0.544
Medical history, *n* (%)						
Prior antiplatelet	40 (7.65)	16 (15.38)	11 (6.08)	6 (5.04)	7 (5.88)	0.011
Prior anticoagulant	25 (4.78)	0 (0.00)	16 (8.84)	0 (0.00)	9 (7.56)	<0.001
Prior statin	28 (5.35)	9 (8.65)	7 (3.87)	6 (5.04)	6 (5.04)	0.381
Antihypertensive	156 (29.83)	51 (49.04)	59 (32.60)	2 (1.68)	44 (36.97)	<0.001
Prior hypoglycemic	72 (13.77)	46 (44.23)	17 (9.39)	6 (5.04)	3 (2.52)	<0.001
Clinical variables						
NIHSS, score	15 (9, 21)	10 (8, 14)	13 (8, 21)	16 (14, 22)	15 (10, 30)	<0.001
ASPECTS, score	7 (6, 8)	8 (8, 10)	8 (7, 9)	7 (6, 8)	6 (5, 7)	<0.001
ONT, minute	185 (133, 240)	192 (80, 259)	172 (133, 225)	186 (148, 215)	186 (138, 243)	0.459
SBP, mmHg	156 (143, 168)	159 (148, 166)	150 (140, 166)	156 (141, 168)	163 (149, 173)	<0.001
DBP, mmHg	87 (79, 99)	93 (81, 95)	85 (79, 94)	86 (79, 97)	89 (79, 109)	0.009
Infarct distribution, *n* (%)						<0.001
Anterior circulation	231 (44.17)	72 (69.23)	68 (37.57)	52 (43.70)	39 (32.77)	
Posterior circulation	59 (11.28)	6 (5.77)	28 (15.47)	6 (5.04)	19 (15.97)	
Anterior and posterior circulation	233 (44.55)	26 (25.00)	85 (46.96)	61 (51.26)	61 (51.26)	
TOAST, *n* (%)						<0.001
LAA	279 (53.35)	78 (75.00)	81 (44.75)	55 (46.22)	65 (54.62)	
CE	154 (29.45%)	14 (13.46)	52 (28.73)	47 (39.50)	41 (34.45)	
SAO	49 (9.37%)	12 (11.54)	28 (15.47)	6 (5.04)	3 (2.52)	
Others	41 (7.84%)	0 (0.00)	20 (11.05)	11 (9.24)	10 (8.40)	
Laboratory data						
BG, mmol/L	6.59 (5.89, 9.27)	6.59 (6.09, 9.65)	6.17 (4.68, 7.74)	6.59 (6.20, 10.91)	6.59 (5.91, 9.59)	<0.001
Homocysteine, μmol/L	17.04 (13.50, 17.04)	17.04 (13.21, 24.58)	17.04 (12.69, 17.04)	17.04 (14.17, 17.29)	17.04 (15.03, 17.04)	0.404
LDL, mmol/L	2.66 (1.98, 2.99)	2.66 (2.25, 3.24)	2.66 (1.90, 2.87)	2.66 (1.66, 3.00)	2.66 (2.60, 2.99)	0.039
HDL, mmol/L	1.10 (0.96, 1.19)	0.99 (0.92, 1.10)	1.10 (1.00, 1.22)	1.10 (0.82, 1.36)	1.10 (1.01, 1.10)	<0.001
Hs-CRP, mg/L	4.40 (0.80, 13.00)	4.95 (0.80, 11.15)	2.20 (0.70, 15.30)	7.10 (1.70, 11.10)	4.00 (1.00, 13.00)	0.541
NLR	6.86 (4.17, 11.32)	5.36 (3.23, 7.43)	6.28 (3.36, 12.18)	7.61 (5.71, 12.23)	7.44 (5.41, 15.32)	<0.001
PIV	676.67 (340.46, 1,010.43)	487.55 (267.55, 779.10)	585.42 (346.90, 1,187.46)	861.57 (410.88, 1,153.99)	792.13 (408.31, 1,115.27)	<0.001
NAR	0.19 (0.15, 0.26)	0.17 (0.11, 0.23)	0.20 (0.14, 0.25)	0.20 (0.18, 0.27)	0.18 (0.15, 0.28)	<0.001

HI1, hemorrhagic infarction type 1; HI2, hemorrhagic infarction type 2; PH1, parenchymal hematoma type 1; PH2, parenchymal hematoma type 2; BMI, Body Mass Index; TIA, transient ischemic attack; CHD, Coronary heart disease; NIHSS, National Institutes of Health Stroke Scale; ONT, Onset-to-Needle Time; SBP, systolic blood pressure; DBP, diastolic blood pressure; TOAST, Trial of Org 10,172 in Acute Stroke Treatment; LAA, large-artery atherosclerosis; CE, cardioembolism; SAO, small-artery occlusion; BG, blood glucose; LDL, low-density lipoprotein; HDL, high-density lipoprotein; Hs-CRP, hyper-sensitive C-reactive protein; NLR, neutrophil-to-lymphocyte ratio; PIV, pan-immune inflammation value; NAR, neutrophil-to-albumin ratio.

### 3.3 Associations of PIV and NAR with HT and its subtypes

As summarized in Table 3, both PIV and NAR showed significant associations with the risk of HT after intravenous thrombolysis in AIS patients. In the fully adjusted model (Model 3), each one-unit increment in log_2_PIV and log_2_NAR was independently associated with an increased risk of HT (OR = 1.69, 95% CI: 1.54–1.85; OR = 2.41, 95% CI: 2.06–3.83; both *p* < 0.001). Quartile analyses further confirmed these associations, with patients in the highest quartile of PIV and NAR exhibiting markedly higher risks of HT compared with those in the lowest quartile (adjusted OR = 15.78, 95% CI: 10.17–24.58; adjusted OR = 10.47, 95% CI: 7.46–14.77; both *P* for trend < 0.001).

**Table 3:** Associations of PIV and NAR with HT and its subtypes.

Outcome	Category	Model 1		Model 2		Model 3	
		OR (95%CI)	*P*	OR (95%CI)	*P*	OR (95%CI)	*P*
Log_2_(PIV)							
HT	Continuous	1.97 (1.83–2.12)	<0.001	1.96 (1.82–2.11)	<0.001	1.69 (1.54–1.85)	<0.001
	Q1	*Ref* (1.00)		*Ref* (1.00)		*Ref* (1.00)	
	Q2	2.22 (1.40–3.62)	0.001	2.27 (1.43–3.69)	0.001	2.10 (1.29–3.41)	0.002
	Q3	4.79 (3.15–7.55)	<0.001	4.65 (3.06–7.34)	<0.001	4.48 (2.89–7.07)	<0.001
	Q4	17.63 (11.94–27.20)	<0.001	17.28 (11.69–26.66)	<0.001	15.78 (10.17–24.58)	<0.001
	*P* for trend	—	<0.001	—	<0.001	—	<0.001
HT Type	HI1	*Ref* (1.00)		*Ref* (1.00)		*Ref* (1.00)	
	HI2	1.22 (1.00–1.49)	0.044	1.23 (1.01–1.50)	0.038	1.20 (0.98–1.48)	0.075
	PH1	1.60 (1.28–1.98)	<0.001	1.60 (1.29–1.99)	<0.001	1.55 (1.25–1.92)	<0.001
	PH2	1.49 (1.20–1.85)	<0.001	1.52 (1.22–1.90)	<0.001	1.47 (1.18–1.83)	<0.001
Log_2_(NAR)							
HT	Continuous	3.61 (3.13–4.16)	<0.001	3.59 (3.12–4.14)	<0.001	2.41 (2.06–2.83)	<0.001
	Q1	*Ref* (1.00)		*Ref* (1.00)		*Ref* (1.00)	
	Q2	1.22 (0.81–1.85)	0.343	1.17 (0.78–1.78)	0.455	1.13 (0.72–1.74)	0.520
	Q3	2.14 (1.48–3.14)	<0.001	2.06 (1.43–3.03)	<0.001	2.00 (1.33–2.94)	<0.001
	Q4	11.10 (8.06–15.65)	<0.001	10.79 (7.83–15.22)	<0.001	10.47 (7.46–14.77)	<0.001
	*P* for trend	—	<0.001	—	<0.001	—	<0.001
HT Type	HI1	*Ref* (1.00)		*Ref* (1.00)		*Ref* (1.00)	
	HI2	1.38 (0.97–1.97)	0.071	1.38 (0.97–1.97)	0.073	1.31 (0.90–1.88)	0.160
	PH1	2.10 (1.40–3.16)	<0.001	2.10 (1.39–3.16)	<0.001	2.00 (1.30–3.04)	<0.001
	PH2	1.90 (1.27–2.85)	0.002	1.91 (1.27–2.85)	0.002	1.79 (1.18–2.67)	0.004

Model 1, unadjusted; Model 2, adjusted for age and sex; and Model 3, further adjusted for smoking, hypertension, atrial fibrillation, prior stroke or TIA, infarct distribution, baseline NIHSS score, ASPECTS, BG, ONT, antiplatelet therapy, anticoagulant therapy, TOAST classification, and LDL. CI, confidence interval; OR, odds ratio; HT, hemorrhagic transformation; HI1, hemorrhagic infarction type 1; HI2, hemorrhagic infarction type 2; PH1, parenchymal hematoma type 1; PH2, parenchymal hematoma type 2; TIA, transient ischemic attack; NIHSS, National Institutes of Health Stroke Scale; ONT, Onset-to-Needle Time; TOAST, Trial of Org 10,172 in Acute Stroke Treatment; BG, blood glucose; PIV, pan-immune inflammation value; NAR, neutrophil-to-albumin ratio; LDL, low-density lipoprotein.

In subtype analyses, PIV and NAR were particularly associated with the more severe forms of HT. Compared with the HI1 group, patients with PH1 had significantly higher levels of both PIV (OR = 1.55, 95% CI: 1.25–1.92, *p* < 0.001) and NAR (OR = 2.00, 95% CI: 1.30–3.04, *p* < 0.001). Similarly, patients with PH2 exhibited increased PIV (OR = 1.47, 95% CI: 1.18–1.83, *p* < 0.001) and NAR (OR = 1.79, 95% CI: 1.18–2.67, *p* = 0.004), suggesting that these inflammatory markers may serve as independent risk factors for severe parenchymal hematomas.

**Figure 5:**
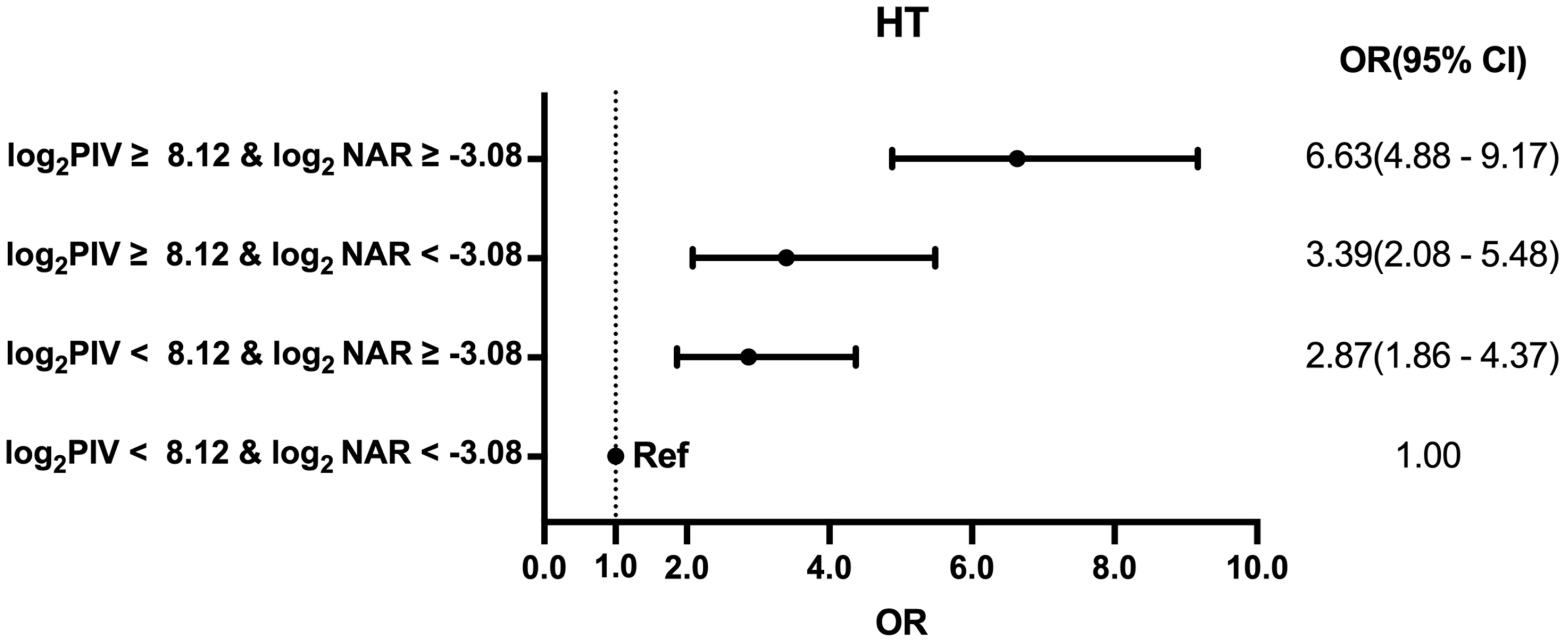
Sensitivity analyses of the joint association between PIV, NAR, and HT in patients with acute ischemic stroke after intravenous thrombolysis. Patients were stratified into four groups according to the median values of log_2_PIV (8.12) and log_2_NAR (−3.08): group 1 (low PIV and low NAR, reference), group 2 (low PIV and high NAR), group 3 (high PIV and low NAR), and group 4 (high PIV and high NAR). Odds ratios were calculated after adjustment for Model 3 covariates. The results demonstrated that elevated levels of both PIV and NAR exerted an additive effect on HT risk, with the highest risk observed in patients in group 4 (OR = 6.63, 95% CI: 4.88–9.17). HT, hemorrhagic transformation; PIV, pan-immune inflammation value; NAR, neutrophil-to-albumin ratio; OR, odds ratio; CI, confidence interval.

To further evaluate the joint predictive value of PIV and NAR, the median values of log_2_PIV (8.12) and log_2_NAR (−3.08) were used as stratification thresholds. Patients were categorized into four groups: group 1 (low PIV and low NAR, reference), group 2 (low PIV and high NAR), group 3 (high PIV and low NAR), and group 4 (high PIV and high NAR). As illustrated in Figure 5, the risk of HT progressively increased across groups 2–4, with the greatest risk observed in group 4 (OR = 6.63, 95% CI: 4.88–9.17). These results indicate an additive effect of elevated PIV and NAR on post-thrombolysis HT.

Interaction analyses provided further evidence of this synergistic effect. On the additive scale, there was a significant interaction between PIV and NAR (RERI = 2.90, 95% CI: 0.89–4.83; AP = 0.44, 95% CI: 0.15–0.63; SI = 2.06, 95% CI: 1.16–3.66). By contrast, no significant interaction was observed on the multiplicative scale (all *P* for interaction > 0.05; Supplementary Tables S1, S2).

Collectively, these findings suggest that both PIV and NAR are independent risk predictors of HT in AIS patients, with their joint elevation conferring a synergistically higher risk, particularly for the severe parenchymal hematoma subtypes.

### 3.4 Restricted cubic splines (RCS) analysis investigating the relationship between PIV, NAR and HT

**Figure 6:**
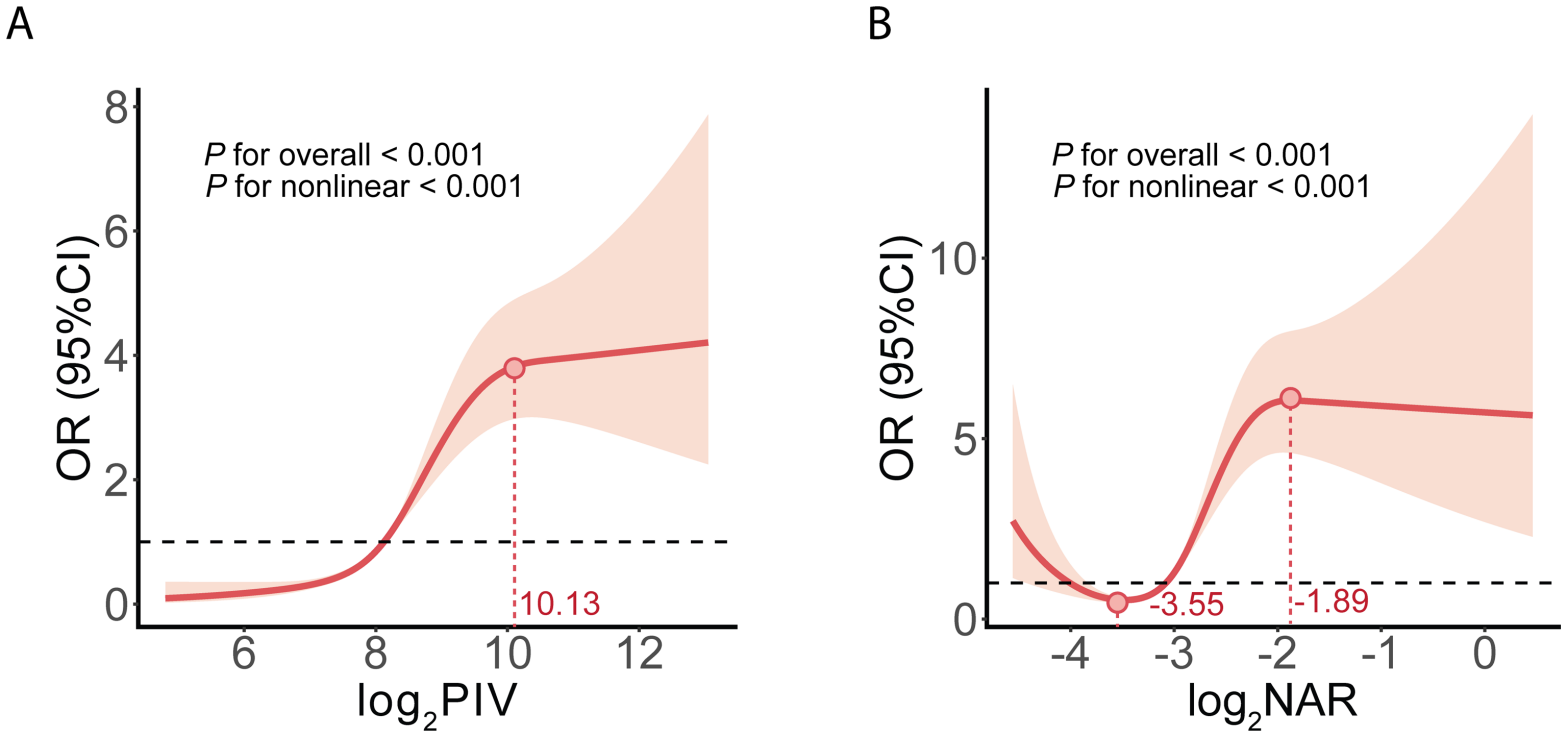
Restricted cubic spline (RCS) curves illustrating the dose-response relationships with HT risk, adjusted for Model 3 covariates. **(A)** Nonlinear association between log2PIV and the OR for HT, showing an inflection point at approximately 10.13. **(B)** Nonlinear association between log2NAR and the OR for HT, highlighting inflection points at approximately -3.55 and -1.89. The solid red lines represent the central estimates, and the shaded areas denote 95% CIs. HT, hemorrhagic transformation; PIV, pan-immune inflammation value; NAR, neutrophil-to-albumin ratio; OR, odds ratio; CI, confidence interval.

To further explore the dose–response relationships between PIV, NAR and HT, we applied RCS models based on multivariable Model 3 adjustment (Figure 6). Both PIV and NAR exhibited significant nonlinear associations with HT (*P*-overall < 0.001; *P*-non-linear < 0.001).

Specifically, the risk of HT increased steeply with rising PIV levels until an inflection point was reached (Supplementary Table S3). Piecewise regression analysis identified this threshold at a log_2_PIV value of 10.13. When PIV ≤ 10.13, the risk of HT increased significantly (OR = 2.56, 95% CI: 2.26–2.91, *p* < 0.001), whereas above this threshold, the association was reversed (OR = 0.62, 95% CI: 0.17–0.34, *p* < 0.001; log-likelihood ratio *p* < 0.001).

Similarly, the association between NAR and HT was nonlinear, with two inflection points at log_2_NAR values of −3.55 and −1.89. Within the interval of −3.55 to −1.89, NAR showed a markedly positive relationship with HT (OR = 7.11, 95% CI: 2.94–106.34, *p* < 0.001). However, outside this range (log_2_NAR < −3.55 or > − 1.89), the relationship weakened or even became inverse (Supplementary Table S3).

### 3.5 Subgroup analysis

We performed stratified multivariate regression analyses to further explore the associations of PIV and NAR with HT in different subgroups, stratified by sex, age, smoking, drinking, hypertension, atrial fibrillation, diabetes mellitus, coronary heart disease, history of prior stroke or TIA, baseline NIHSS score, and TOAST classification.

**Figure 7:**
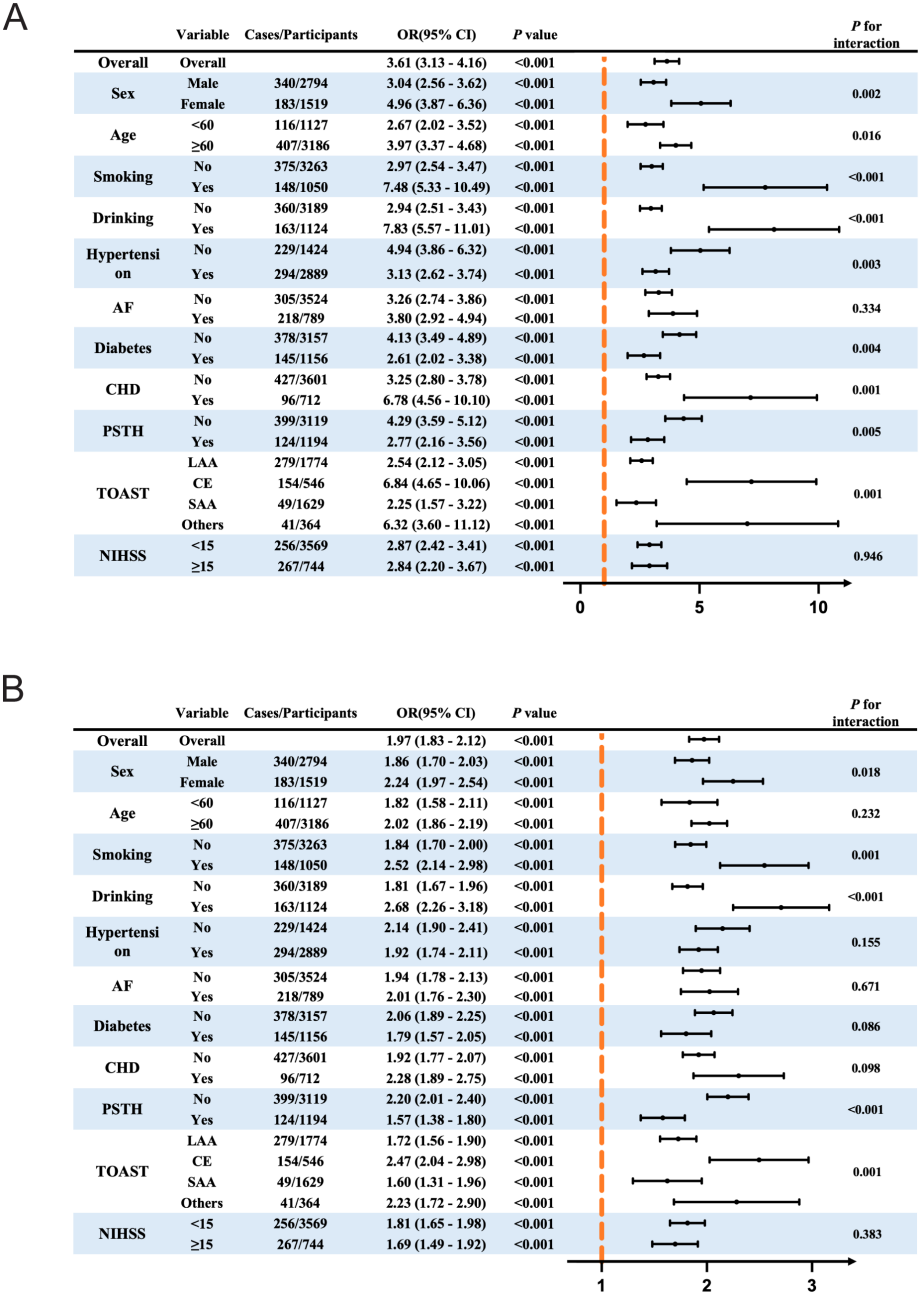
Subgroup analyses examining the relationship between PIV, NAR, and HT in AIS, adjusted for Model 3 covariates. **(A)** Subgroup analyses examining the relationship between PIV and HT in AIS. **(B)** Subgroup analyses examining the relationship between NAR and HT in AIS. HT, hemorrhagic transformation; AF, atrial fibrillation; CHD, Coronary heart disease; PSTH, History of prior stroke or transient ischemic attack; NIHSS, National Institutes of Health Stroke Scale; TOAST, Trial of Org 10,172 in Acute Stroke Treatment; LAA, large-artery atherosclerosis; CE, cardioembolism; SAA, Small-artery occlusion; PIV, pan-immune inflammation value; NAR, neutrophil-to-albumin ratio; CI, confidence interval.

As shown in Figure 7, both PIV and NAR were significantly associated with an increased risk of HT in patients with AIS, and the positive associations were generally consistent across most subgroups (*P* for interaction > 0.05 in most cases). However, several significant interactions were identified. Specifically: PIV exhibited significant interactions with sex (*p* = 0.018), smoking (*p* = 0.001), and drinking status (*p* < 0.001) (Figure 7A). NAR showed significant interactions with sex (*p* = 0.002), age (*p* = 0.016), smoking (*p* < 0.001), drinking (*p* < 0.001), hypertension (*p* = 0.003), diabetes mellitus (*p* = 0.004), coronary heart disease (*p* = 0.001), and TOAST classification (*p* = 0.001) (Figure 7B).

The stratified analyses of the joint association between log_2_PIV and log_2_NAR with HT are presented in Supplementary Table S4. Patients were categorized into four groups according to the median values of log_2_PIV (8.12) and log_2_NAR (−3.08): group 1 (low PIV and low NAR, reference), group 2 (low PIV and high NAR), group 3 (high PIV and low NAR), and group 4 (high PIV and high NAR). Compared with the reference group, patients in group 4 exhibited a markedly higher risk of HT across almost all subgroups. The combined effect of elevated PIV and NAR was particularly pronounced in females (OR = 17.86, 95% CI: 10.00–31.87), younger patients <60 years (OR = 10.00, 95% CI: 5.38–18.58), smokers (OR = 17.90, 95% CI: 8.97–35.70), drinkers (OR = 17.55, 95% CI: 8.82–34.92), and patients with cardioembolic stroke (CE) according to TOAST classification (OR = 44.10, 95% CI: 15.92–122.16).

These findings suggest that while the relationships of PIV and NAR with HT are broadly consistent, certain subgroups—particularly those defined by sex, lifestyle factors (smoking and alcohol consumption), comorbidities (diabetes, hypertension, CHD), and stroke etiology (TOAST classification)—may be more susceptible to HT when both systemic immune-inflammatory and nutritional status imbalances coexist.

### 3.6 Prognostic value of PIV, NAR, and combined of them in AIS patients

**Figure 8:**
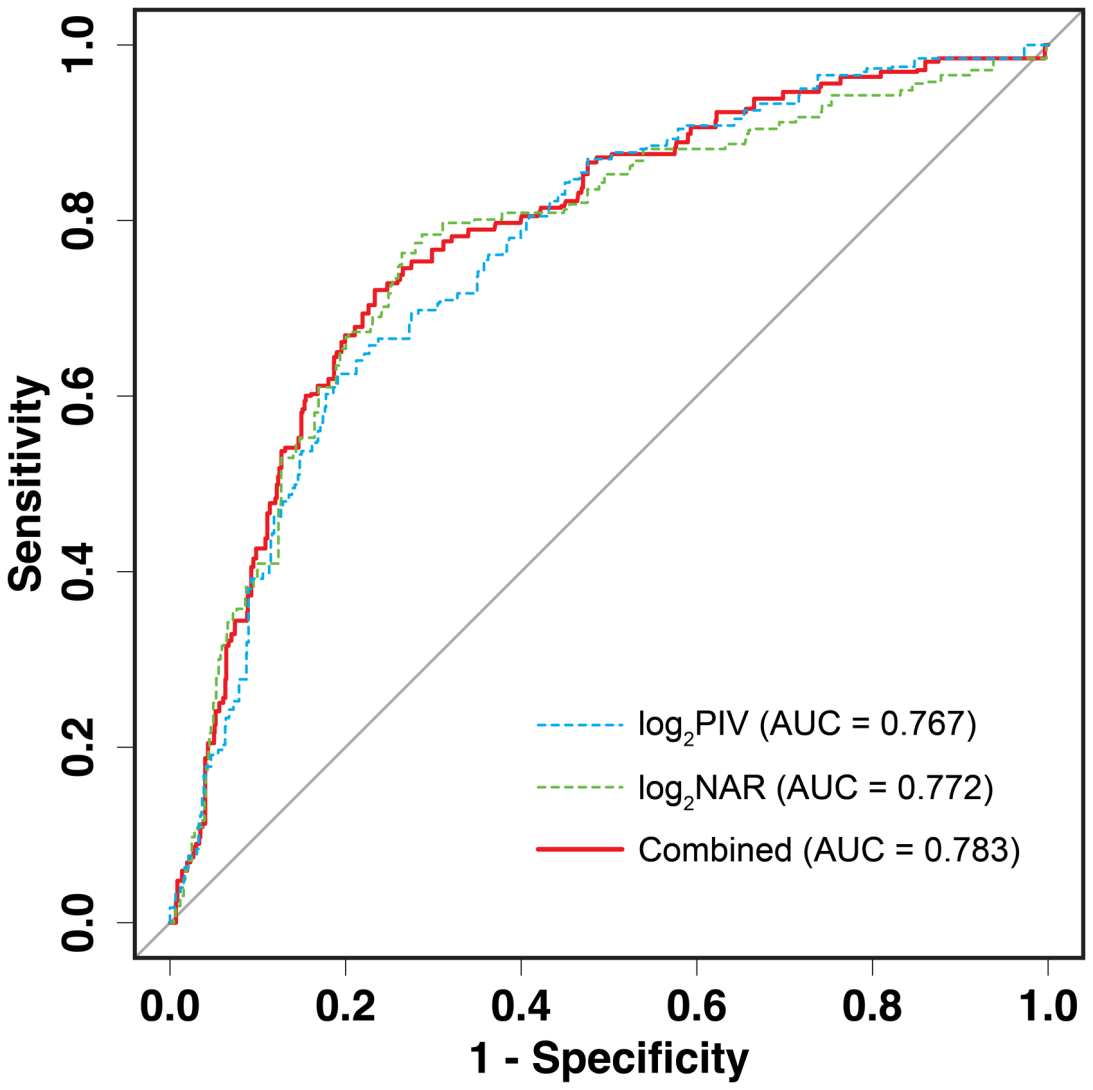
ROC curve analysis of PIV, NAR, and their combination for predicting HT after IVT. ROC curves illustrating the predictive performance of log_2_PIV, log_2_NAR, and their combined model for HT following IVT in patients with acute ischemic stroke. The combined model demonstrated the highest discriminative ability compared with either biomarker alone. HT, hemorrhagic transformation; IVT, intravenous thrombolysis; PIV, pan-immune inflammation value; NAR, neutrophil-to-albumin ratio; AUC, area under curve.

ROC curve analyses were performed to evaluate the predictive value of PIV, NAR, and their combination for HT after IVT. As shown in Figure 8, both log_2_PIV (AUC = 0.767; 95% CI, 0.746–0.788) and log_2_NAR (AUC = 0.772; 95% CI, 0.750–0.795) demonstrated good discriminative ability for post-thrombolysis HT. Importantly, the combined model incorporating both markers significantly improved predictive accuracy, yielding an AUC of 0.783 (95% CI, 0.761–0.804; *p* < 0.001).

Comparative analyses revealed that the combined model was superior to PIV alone (AUC: 0.783 vs. 0.767, *p* < 0.001) and to NAR alone (AUC: 0.783 vs. 0.772, *p* = 0.017). Based on the maximum Youden index, the optimal cutoff values were 9.01 for log_2_PIV, −2.76 for log_2_NAR, and 0.13 for the combined model, with corresponding sensitivity and specificity values summarized in Supplementary Table S5. Furthermore, the incremental predictive value of PIV, NAR, and their combination beyond the basic model was evaluated using the NRI and IDI. As shown in Supplementary Table S6, adding PIV or NAR to the basic model significantly improved risk reclassification and discrimination for HT. Notably, the combined model provided the greatest improvement (NRI: 0.256, 95% CI: 0.211–0.301; IDI: 0.105, 95% CI: 0.092–0.118; both *p* < 0.001).

Collectively, these findings indicate that combining PIV and NAR enhances predictive performance beyond either biomarker alone, supporting their use as a complementary risk assessment tool for early identification of HT in AIS patients receiving IVT.

## 4 Discussion

In this dual-center retrospective cohort study, we demonstrated that elevated PIV and NAR, both individually and in combination, were independently associated with an increased risk of HT following intravenous thrombolysis in AIS patients. Importantly, the joint elevation of these biomarkers demonstrated an additive association with HT risk, particularly for severe subtypes such as parenchymal hematoma (PH1 and PH2). Moreover, their combination significantly improved predictive accuracy, highlighting their potential utility as readily accessible, multidimensional biomarkers for individualized risk stratification.

Previous studies have consistently reported that HT represents one of the most devastating complications of thrombolysis, associated with poor functional outcomes and increased mortality (4, 36). However, reported incidences vary widely across cohorts, ranging from 10 to 48% (37). In our large sample (*n* = 4,313), the incidence of HT was 12.1%, consistent with rates observed in randomized controlled trials, thereby reinforcing the robustness of our findings. Notably, we extended prior research by systematically analyzing HT subtypes. While hemorrhagic infarction (HI) is often clinically silent, parenchymal hematoma (PH) is strongly linked to an increased risk of neurological deterioration and death, with case fatality rates approaching 50% (29, 38, 39). We observed that both PIV and NAR were particularly associated with PH1 and PH2, underscoring their relevance in identifying patients at risk for the most severe HT phenotypes.

Neuroinflammation is increasingly recognized as a potential key contributor to BBB disruption and secondary brain injury, leading to the conceptual expansion from the classical “neurovascular unit” to the broader “neurovascular–immune network” (40). PIV, which integrates neutrophils, monocytes, platelets, and lymphocytes into a single index, reflects systemic immune-inflammatory status and the imbalance of immune networks. Following AIS, ischemia and hypoxia are thought to trigger robust activation of innate and adaptive immune responses, often accompanied by the release of proinflammatory mediators including TNF-*α*, reactive oxygen species, and MMP-9 (41–43). These mediators are known to have the capacity to degrade extracellular matrix components, impair vascular integrity, and increase BBB permeability, processes that may facilitate HT. In parallel, excessive inflammatory activation may lead to stroke-induced immunosuppression syndrome (SIDS), characterized by lymphocyte and monocyte depletion, immune cell apoptosis, and increased vulnerability to infection (44, 45). Platelets may further contribute by potentially promoting hypercoagulability and amplifying systemic inflammation via cytokines such as thromboxane A2 (46). Together, we hypothesize that these interacting pathways could help explain why elevated PIV, as a composite measure of systemic inflammation and immune dysregulation, was strongly associated with HT risk, particularly PH.

Interestingly, our RCS analysis revealed a non-linear threshold relationship between PIV and HT. Specifically, risk increased steeply up to a log_2_PIV value of 10.13 but declined beyond this threshold. Similar threshold or “plateau” phenomena have been observed in oncology studies, where excessively high PIV levels paradoxically lost predictive value for poor outcomes (47). One possible explanation is that very high PIV values may reflect compensatory immunological adaptations, including relative neutrophil depletion or lymphocyte recovery, which require further mechanistic exploration.

The NAR also emerged as a novel and clinically meaningful biomarker. Neutrophils contribute to oxidative stress, proteolytic damage, and vascular dysfunction, while albumin is considered to possess protective effects through antioxidative, anti-inflammatory, and endothelial-stabilizing properties (48, 49). Elevated NAR, therefore, likely reflects heightened systemic inflammation in the context of impaired nutritional and vascular resilience. Prior studies have linked high NAR to adverse outcomes in traumatic brain injury, subarachnoid hemorrhage, and cardiovascular disease (28, 50, 51). In our cohort, high NAR independently predicted HT, especially PH1 and PH2. Notably, RCS analyses revealed a log-shaped association, with protective effects at very low levels and sharply increased risk in the −3.55 to −1.89 log_2_NAR range. A similar log-shaped relationship has been reported in cardiovascular populations, where both extremely low and extremely high NAR values were associated with adverse outcomes (52). These findings support a threshold-dependent relationship, wherein adequate albumin and moderate immune activity may correlate with vascular protection, whereas excessive neutrophil-driven inflammation might overwhelm compensatory mechanisms, potentially contributing to BBB breakdown and HT (53–55).

The additive interaction between PIV and NAR highlights the synergistic role of immune-inflammatory activation and nutritional depletion. Patients with simultaneous elevations exhibited a more than six-fold increased HT risk, with particularly strong associations observed in females, younger patients, smokers, drinkers, and those with cardioembolic stroke. Several mechanisms may underlie these subgroup differences. For instance, sex-related immune dimorphism, with women generally exhibiting stronger inflammatory responses, may increase vulnerability to HT under conditions of systemic immune activation (56). Younger patients often have a more reactive immune system, which could amplify inflammatory cascades once triggered by ischemia and thrombolysis (57). Smoking and alcohol consumption are well-established risk factors for endothelial dysfunction and oxidative stress, both of which are thought to exacerbate BBB disruption (58, 59). Finally, cardioembolic stroke is typically associated with larger infarct volumes and greater reperfusion injury, conditions that may create an environment susceptible to inflammation-mediated hemorrhagic conversion (60). These findings suggest that certain populations may be especially vulnerable to combined immune and nutritional imbalance, warranting tailored monitoring and intervention strategies.

This study has several strengths, including its large dual-center design, detailed classification of HT subtypes, and comprehensive subgroup and sensitivity analyses that reinforced the robustness of our findings. To our knowledge, this is the first study to systematically examine both independent and joint associations of PIV and NAR with HT after thrombolysis, with particular attention to clinically relevant subtypes. These features enhance the translational value of our work and highlight the potential utility of PIV and NAR as simple, bedside-accessible biomarkers. Nonetheless, several limitations should be acknowledged. First, the retrospective design precludes causal inference and introduces potential confounding. Second, PIV and NAR were measured only at baseline; dynamic changes over time could provide additional prognostic information. Third, the study population was restricted to two centers in China, which may limit generalizability. Finally, our findings are based on patients treated exclusively with alteplase; validation is needed in populations receiving tenecteplase, which is increasingly adopted worldwide.

## 5 Conclusion

In conclusion, our study provides novel evidence that PIV and NAR are independent and complementary predictors of HT after intravenous thrombolysis in AIS patients. Their combination offers enhanced predictive performance and likely reflects the immunological interplay between systemic inflammation and nutritional status. Given their accessibility and cost-effectiveness, integrating PIV and NAR into pre-thrombolysis evaluation may help clinicians identify high-risk patients, optimize therapeutic strategies, and improve outcomes. Future prospective and mechanistic studies are warranted to validate these findings and further elucidate the underlying immunological pathways.

## Data Availability

The raw data supporting the conclusions of this article will be made available by the authors, without undue reservation.
