# Supplementary material for: Pan-immune inflammation value and neutrophil-to-albumin ratio predict hemorrhagic transformation after intravenous thrombolysis in acute ischemic stroke: a dual-center cohort study
Source: Front Nutr. 2026 May 18;13:1724969. doi: 10.3389/fnut.2026.1724969 (PMC13224339; doi:10.3389/fnut.2026.1724969)
Supplement: Supplementary file 1 [file Data_Sheet_1.pdf]

Pan-immune inflammation value and neutrophil-to-albumin ratio predict hemorrhagic transformation after intravenous thrombolysis in acute ischemic stroke: a dual-center cohort study

Supplementary Materials:

Table S1. Assessment of the additive interaction between log_2PIV and log_2NAR on HT.

Groups	aOR (95% CI)	RERI (95% CI)	AP (95% CI)	SI (95% CI)
low PIV & low NAR	1.0 (ref)			
low PIV & high NAR	1.33 (0.78−2.28)	2.90 (0.89−4.83)	0.44 (0.15−0.63)	2.06 (1.16−3.66)
high PIV & low NAR	3.40 (2.10−5.51)			
high PIV & high NAR	6.63 (4.84−9.09)			

Patients were stratified into four groups according to the median values of log_2PIV (8.12) and log_2NAR (−3.08): group 1 (low PIV & low NAR, reference), group 2 (low PIV & high NAR), group 3 (high PIV & low NAR), and group 4 (high PIV & high NAR). Odds ratios were calculated after adjustment for Model 3 covariates. Note: RERI: Relative excess risk due to interaction; AP: attributable proportion; SI: synergy index

Table S2. Assessment of the multiplicative interaction between log_2PIV and log_2NAR on HT.

Groups	aOR (95% CI)	*P*	*P* interaction
log_2PIV			
log_2NAR < median (-3.08)	2.05(1.52−2.77)	<0.001	0.119
log_2NAR ≥ median (-3.08)	1.63 (1.46−1.84)	<0.001	
log_2NAR			
log_2PIV < median (8.12)	1.71 (0.94−3.12)	0.081	0.100
log_2PIV ≥ median (8.12)	1.88 (1.53−2.32)	<0.001	

Table S3. Analysis of threshold effects for HT.

	Adjusted OR (95% CI)	*P*-value
**log_2PIV**		
Fitting by linear regression model	1.82 (1.68-1.98)	<0.001
Fitting by two-piecewise linear regression model		
Inflection point	10.13	
≤ 10.13	2.56 (2.26-2.91)	<0.001
> 10.13	0.62 (0.17-0.34)	<0.001
Log-likelihood ratio		<0.001
**log_2NAR**		
Fitting by linear regression model	2.66 (2.28-3.11)	<0.001
Fitting by two-piecewise linear regression model		
Inflection point	-3.55，-1.89	
< -3.55	0.19 (0.08- 0.49)	<0.001
-3.55 ~ -1.89	7.11 (12.94-106.34)	<0.001
> -1.89	0.22 (0.01-0.07)	<0.001
Log-likelihood ratio		<0.001

Threshold effect analyses: all models were adjusted for Model 3 covariates. Abbreviations: CI: confidence interval; OR: odds ratio; HT: hemorrhagic transformation; PIV: pan-immune inflammation value; NAR: neutrophil-to-albumin ratio.

Table S4. Stratified analyses of the joint association between log_2PIV and log_2NAR with HT.

Variables	low PIV & low NAR	low PIV & high NAR	high PIV & low NAR	high PIV & high NAR	*P* for interaction
Sex					
Male	1.00 (ref)	1.40 (0.74-2.64)	3.07 (1.93-4.88)	6.50 (4.66-9.07)	<0.001
Female	1.00 (ref)	3.19 (1.43-7.12)	1.23 (0.34-4.38)	17.86 (10.00-31.87)	
Age					
< 60 years	1.00 (ref)	1.03 (0.33-3.23)	7.82 (3.42-17.88)	10.00 (5.38-18.58)	0.005
≥ 60 years	1.00 (ref)	2.24 (1.31-3.83)	2.07 (1.25-3.43)	8.58 (6.21-11.86)	
Smoking					
No	1.00 (ref)	1.45 (0.84-2.50)	2.88 (1.82-4.54)	7.23 (5.26-9.93)	0.012
Yes	1.00 (ref)	4.61 (1.59-13.41)	2.32 (0.70-7.70)	17.90 (8.97-35.70)	
Drinking					
No	1.00 (ref)	1.53 (0.89-2.63)	3.20 (2.03-5.06)	7.15 (5.20-9.84)	0.001
Yes	1.00 (ref)	3.67 (1.27-10.61)	1.66 (0.50-5.49)	17.55 (8.82-34.92)	
Hypertension					
No	1.00 (ref)	1.94 (0.95-3.95)	4.16 (2.07-8.37)	12.00 (7.50-19.21)	0.393

Yes	1.00 (ref)	1.65 (0.84-3.21)	2.44 (1.42-4.18)	7.46 (5.19-10.72)	
AF					
No	1.00 (ref)	2.04 (1.16-3.56)	2.68 (1.60-4.48)	7.06 (5.00-9.97)	0.051
Yes	1.00 (ref)	1.26 (0.48-3.31)	3.36 (1.54-7.33)	12.58 (7.40-21.38)	
Diabetes					
No	1.00 (ref)	2.68 (1.54-4.66)	3.05 (1.82-5.10)	9.65 (6.76-13.78)	0.084
Yes	1.00 (ref)	0.64 (0.22-1.89)	2.65 (1.23-5.68)	7.92 (4.86-12.91)	
CHD					
No	1.00 (ref)	2.02 (1.22-3.35)	3.22 (2.06-5.03)	7.66 (5.67-10.35)	<0.001
Yes	1.00 (ref)	1.24 (0.22-6.91)	1.85 (0.41-8.41)	24.66 (8.89-68.36)	
PSTH					
No	1.00 (ref)	1.81 (1.07-3.07)	2.63 (1.59-4.34)	7.98 (5.83-10.91)	0.346
Yes	1.00 (ref)	1.99 (0.59-6.72)	4.78 (1.94-11.75)	14.83 (7.12-30.88)	
TOAST					
LAA	1.00 (ref)	2.14 (1.20-3.79)	1.90 (1.07-3.40)	4.59 (3.24-6.50)	<0.001
CE	1.00 (ref)	6.10 (1.65-22.56)	12.59 (3.66-43.34)	44.10 (15.92-122.16)	
SAA	1.00 (ref)	0.00 (0.00-Inf)	5.46 (2.17-13.75)	6.42 (2.92-14.12)	
Others	1.00 (ref)	0.00 (0.00-Inf)	0.00 (0.00-Inf)	18.73 (4.43-79.27)	
NIHSS					
< 15	1.00 (ref)	1.34 (0.73-2.47)	2.28 (1.35-3.86)	6.39 (4.57-8.93)	0.494
≥ 15	1.00 (ref)	3.01 (1.24-7.32)	3.04 (1.36-6.79)	9.50 (5.19-17.40)	

Patients were stratified into four groups according to the median values of log_2PIV (8.12) and log_2NAR (−3.08): group 1 (low PIV & low NAR, reference), group 2 (low PIV & high NAR), group 3 (high PIV & low NAR), and group 4 (high PIV & high NAR). Odds ratios (ORs) with 95% confidence intervals (CIs) were calculated after adjustment for Model 3 covariates. Abbreviations: HT: hemorrhagic transformation; AF: atrial fibrillation; CHD: Coronary heart disease; PSTH: History of prior stroke or transient ischemic attack; NIHSS: National Institutes of Health Stroke Scale; TOAST: Trial of Org 10,172 in Acute Stroke Treatment; LAA: large-artery atherosclerosis; CE: cardioembolism; SAA: Small-artery occlusion; PIV: pan-immune inflammation value; NAR: neutrophil-to-albumin ratio; CI: confidence interval.

Table S5. Diagnostic performance of log_2PIV, log_2NAR, and their combination for predicting HT after IVT.

	**AUC (95%CI)**	***P*-Value**	**Optimal cut-off**	**Sensitivity**	**Specificity**
**HT**					
log_2PIV	0.767 (0.746-0.788)	<0.001	9.01	0.63	0.81
log_2NAR	0.772 (0.750-0.795)	<0.001	-2.76	0.76	0.74
Combined	0.783 (0.761-0.804)	<0.001	0.13	0.72	0.77

Cutoff values were determined according to the maximum Youden index. Sensitivity and specificity values are presented for each biomarker and the combined model. Abbreviations: HT: hemorrhagic transformation; IVT: intravenous thrombolysis; ROC: receiver operating characteristic; AUC: area under the curve; CI: confidence interval; OR: odds ratio; PIV: pan-immune inflammation value; NAR: neutrophil-to-albumin ratio.

Table S6. Incremental predictive value of PIV, NAR, and their combination beyond the basic model for HT

**Models**	NRI (95% CI)	*P* Value	IDI (95% CI)	*P* Value
**HT**				
Basic model	Ref		Ref	
Basic model+ log_2PIV	0.198(0.153-0.242)	<0.001	0.072(0.061-0.084)	<0.001
Basic model+ log_2NAR	0.149(0.107-0.191)	<0.001	0.057(0.047-0.068)	<0.001
Basic model+ log_2PIV+ log_2NAR	0.256(0.211-0.301)	<0.001	0.105(0.092-0.118)	<0.001

Abbreviations: NRI: net reclassification index; IDI: integrated discrimination improvement; CI: confidence interval; HT: hemorrhagic transformation; PIV: pan-immune inflammation value; NAR: neutrophil-to-albumin ratio; Basic model included age, sex, smoking, hypertension, atrial fibrillation, prior stroke or TIA, infarct distribution, baseline NIHSS score, ASPECTS, BG, ONT, antiplatelet therapy, anticoagulant therapy, TOAST classification, and LDL.
